# Sanitary condition and its microbiological quality of improved water sources in the Southern Region of Ethiopia

**DOI:** 10.1007/s10661-020-08297-z

**Published:** 2020-04-30

**Authors:** Tsigereda Assefa Alemayehu, Abel Weldetinsae, Daniel Abera Dinssa, Firehiwot Abera Derra, Tesfaye Legese Bedada, Yosef Beyene Asefa, Sisay Derso Mengesha, Zinabu Assefa Alemu, Melaku Gizaw Serte, Kirubel Tesfaye Teklu, Mesay Getachew Woldegabriel, Moa Abate Kenea, Harold van den Berg, Ana Maria de Roda Husman

**Affiliations:** 1 Ethiopian Public Health Institute, Addis Ababa, Ethiopia; 2 National Institute for Public Health and the Environment, Bilthoven, The Netherlands

**Keywords:** *E. coli*, Enterococci, Risk priority matrix

## Abstract

In the Southern Nations, Nationalities, and Peoples’ Region of Ethiopia, improved water is the main source of water for household purposes. Access to improved water closer to their homes benefits the community in many ways. It improves their health status, saves their time and energy, and improves their productivity in jobs and education they are engaged in. However, due to natural and human activities, improved water sources do not always deliver good quality of water. It can be contaminated by different pathogenic microorganisms and chemicals. The result indicated that 44.7% and 50.9% of the samples were contaminated with *Escherichia coli* and enterococci respectively, and from the sanitary condition survey, 57.6% of the water sources exhibited from intermediate- to very high-risk level. And the risk priority matrix identifies 95 (27.9%) samples with high risk and 54 (15.9%) of the samples with very high risk. The main risks identified at those unsafe water sources were that the drainage canals were blocked with mud, grass, leaves, and stones; animals drinking the overflow water and grazing in the proximity of water sources and feces such as cow dung were observed; inadequate protection of water sources such as absence of fences and diversion ditches; and stagnant water near the source. The study conducted in the Southern Nations, Nationalities, and Peoples’ Region has clearly indicated that people may be at risk of being exposed to pathogens in half of the improved water sources when used for drinking based on the microbial indicator data or the sanitary inspection risk score. Though no correlation resulted from water quality and sanitary condition of sources, the risk priority matrix did enable prioritization of 54 very high-risk-level water sources for urgent targeted interventions from a total of 340 improved drinking water sources. From this, targeted interventions, improving water management practices, identifying and implementing effective water treatment options, providing sustainable energy sources for the supply of continuous water, and implementing climate resilience water safety planning, are recommended.

## Background

Worldwide, 159 million people in 2015 still collect drinking water directly from surface water sources, of whom 58% lived in sub-Saharan Africa (WHO/UNICEF 2017). In many low-income countries including those in Africa, it may be difficult to deliver tap water to each and every household in rural areas. The development of water infrastructure is very expensive because fewer people are serviced over large distance of piping. Opting for improved water supplies has been at the attention of Ethiopian Government because it benefits the community in many ways, such as access to water closer to their homes which improves their health status, saves their time and energy, and improves their productivity in jobs and education they are engaged in. The present improved water sources may not always deliver good quality of water. It can be contaminated by chemicals and pathogenic microorganisms.

In Ethiopia, about 75% of health problem is communicable disease associated with unsafe and inadequate water and poor human excreta disposal (Tabor et al. 2011). Waterborne infectious diseases are caused by a variety of microbial agents such as viruses, bacteria, protozoa, and helminths. Some of the diseases associated with those agents include cholera, gastroenteritis, infectious hepatitis, diarrhea, typhoid fever, giardiasis, poliomyelitis, and ascariasis (Ashbolt 2004). Cholera is one of the worst waterborne diseases caused by the bacterium *Vibriocholerae*, responsible for acute watery diarrhea and lead to illness and death to human beings in many parts of the world. In June 2006, in Oromia region of Ethiopia, the outbreak of acute watery diarrhea was reported, resulting in more than 1900 cases in mid-September (Bartels et al. 2010). This disease spread to the different Ethiopian regions of Amhara, Tigray, and Southern Nations, Nationalities, Peoples’ Region (SNNPR) (Bartels et al. 2010). Since that time, this disease relapses every year especially in the rainy season and continues to be public health concern.

Studies indicate that improper management of drinking water sources, such as inadequate maintenance, poor sanitary practice, and improper site selection are the major factors that lead to the contamination of drinking water. A water quality monitoring performed by Howard et al. (2003a) in Uganda on ground water justified that the concentration of thermotolerant and fecal streptococci increased in the rainy season due to poor construction of wellheads and sanitary seals, inadequate fences, and erosion of backfill area. Lack of appropriate management of human excreta disposal system is another route for the entrance of pathogenic microorganisms into ground and surface water (Howard et al., 2003a). Ground water can be contaminated by fecal matter drawn from the underlying aquifer (Graham & Polizzotto 2013). It is supported by the study conducted in Marondera district, Zimbabwe, by Dzwairo et al. (2006) showing that ground water is contaminated by total and fecal coliforms due to leach of contaminants from pit latrines. Agricultural activities are also another source of water pollution. Manure applied to land as a fertilizer can be washed away from farm fields during the rainy season and join the water sources which can serve as microbial contaminant.

In the Southern Region of Ethiopia, drinking water is obtained from improved sources such as caped springs, protected shallow wells, and boreholes. Studies have shown that water quality in this region is more deprived compared with that in other parts of Ethiopia. Rapid water quality assessment was performed by Tadesse et al. (2010) on improved water sources from 2004 to 2005; the SNNPR water sources had the lowest microbial water quality than other regions of Ethiopia. From that time to present, there is no organized water quality assessment that can cover most areas of this region. Therefore, the main objectives of this survey were to assess the quality of water and factors that contributes to the contamination of water and to identify the water sources that are at higher risk and priorities that need intervention.

## Materials and methods

### Study area

**Fig. 1:**
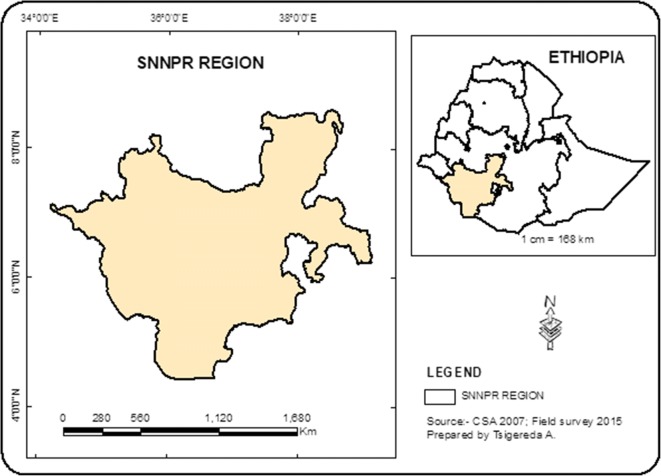
Map of SNNPR

SNNPR (Fig. 1) is one of the nine regions in Ethiopia and lies in the southern part of the country. It is the largest region covering an area of 112,323.19 km^2^. It lies between 40.43–80.58 North latitude and 340.88–390.14 East longitude. The elevation ranges from 376 to 4, 207 m above sea level. It is bordered by national boundaries in the North-West Gambela Regional state and surrounded by Oromiya Regional State in the North-East directions. It extends its boundaries to Kenya in the South and the Republic of Sudan in the South-West. About 56% of the total area of the state is found below 1500-m elevation, which is categorized largely as hottest low land. The rest 44% is found in the temperate climatic zone. The rainfall intensity and duration varies from place to place within the region. It decreases from West and North-West to South-Eastwards. The mean annual rainfall ranged from 400 to 2200 mm, mainly in the months of February to March. Of the regional population, 90% engage in farming and pastoral system.

### Sampling design

The survey was conducted from October 2014 to June 2015. It was carried out by taking water samples and at the same time performing visual inspection of the sanitary condition of the water source, the so-called sanitary inspection (WHO 2012). The water samples for the study purposes were taken from improved water sources including boreholes, caped springs, and protected shallow wells. The community used these water sources for drinking and other household purposes. Sampling sites were selected based on a stratified random sampling method described by Howard et al. (2003a). The sampling covered all different geographical areas and climate conditions in SNNPR. This survey assessed the sanitary condition of water sources and the microbial water quality. During sample collection, it was attempted to collect additional data related to the water quality and sanitary inspection including if the water is treated from the source and the type of treatment practiced.

### Microbial analysis

Microbial water quality was assessed by analyzing indicator bacteria *E. coli* and enterococci. These bacteria are regarded as the most common and reliable microbial indicators exclusively fecal in origin that shows contamination of water with fecal matter (Howard et al., 2003a). Enterococci have been used to augment testing for *E. coli* because most of the enterococci species are also fecal in origin and survive longer than *E. coli* (WHO 2008). For the purpose of bacterial analyses, water samples were collected by sterilized glass bottles placed in an ice-packed container to maintain the temperature around 4 °C and transported to the nearest zonal public health laboratories. All samples were analyzed within 12 h of collection and colony-forming units of *E. coli* and enterococci. It was enumerated by taking 100-ml aliquots of the water sample based on Ethiopian Standard Methods ES ISO 9308-1:^2001^ and ES ISO 7899-2:^2005^ respectively.

### Sanitary inspection

Sanitary inspection was performed during water sample collection for each sampling site by following standard format described by WHO (2012). Different sanitary inspection forms representing three types of water sources, borehole, caped spring, and protected shallow wells, were prepared. These forms consist of a set of questions with “yes” or “no’ answers to help in finding the most important factors that contribute to the contamination of water. Some of the questions indicate structural deficiencies in the construction of concrete walls, distance between the water source and latrine, and proper fencing to avoid the entrance of animals and people closer to the water source. The number of “yes” answers indicates risk of the entry of contaminants into the water source and the number of “no” answers indicates no or negligible risk of contamination of the given water source. By summing all “yes” answers in the given filled form, the total number of risks of contamination was obtained at 0 to 10 score scale. Answers were reevaluated into different risk categories as low, intermediate, high, and very high risk.

### Combined sanitary inspection and water quality analysis

To find out water sources that need more attention and to give priorities for the intervention, a risk priority matrix was prepared by coupling sanitary condition survey with water quality test results. The risk assessment and the recommended remedial action prioritization are based on the microbiological load level and sanitary inspection risk score.

### Data analysis

IBM SPSS 20 for windows software was used to analyze descriptive statistics such as percentages, ranges, geometric mean, and frequency. To find if there is any significant association between sanitary risk score and microbial contamination of the water, Spearman’s rank correlation was applied.

## Results

A total of 340 water samples were collected of which 88 from boreholes, 130 from caped springs, and 122 from protected shallow wells. The result of microbial analysis and sanitary inspection of the water source is presented in terms of percentages of water samples in Tables 1 and 2. And risk priority matrix by coupling water quality and sanitary inspection is presented in Table 3.

**Table 1:** Percentage (%) of water samples contaminated with *E. coli* and/or enterococci by water source type

Indicator organisms		Risk categories and their respective bacterial load in CFU/ml of water				
		No risk	Low risk	Intermediate risk	High risk	Very high risk
*E. coli*	Water source	< 1	1–10	10–100	100–1000	> 1000
		No (%)	No (%)	No (%)	No (%)	No (%)
	BH	53 (60.2)	10 (11.4)	14 (15.9)	7 (8)	4 (4.5)
	CS	60 (46.1)	20 (15.4)	27 (20.8)	14 (10.8)	9 (6.9)
	SW	75 (61.5)	12 (9.8)	18 (14.8)	13 (10.6)	4 (3.3)
	Total	188 (55.3)	42 (12.4)	59 (17.4)	34 (10)	17 (5)
Enterococci	BH	51 (58)	16 (18.2)	12 (13.6)	9 (10.2)	0 (0.0)
	CS	46 (35.4)	33 (25.4)	27 (20.7)	20 (15.4)	4 (3.1)
	SW	70 (57.4)	22 (18.0)	19 (15.6)	10 (8.2)	1 (0.8)
	Total	167 (49.1)	71 (20.9)	58 (17.1)	39 (11.5)	5 (1.5)

*BH* borehole, *CS* caped spring, *SW* shallow well

**Table 2:** Sanitary inspection risk score for each type of water source

Sanitary inspection risk score				
	Low 0–2	Intermediate 3–5	High 6–8	Very high > 8
Water source	No (%)	No (%)	No (%)	No (%)
BH	49 (55.7)	31 (35.2)	8 (9.1)	0 (0.0)
CS	43 (33.1)	51 (39.2)	32 (24.6)	4 (3.1)
SW	52 (42.6)	55 (41.5)	14 (11.5)	1 (0.8)
Total	144 (42.2)	137 (40.3)	54 (15.9)	5 (1.5)

*BH* borehole, *CS* caped spring, *SW* shallow well

**Table 3:**
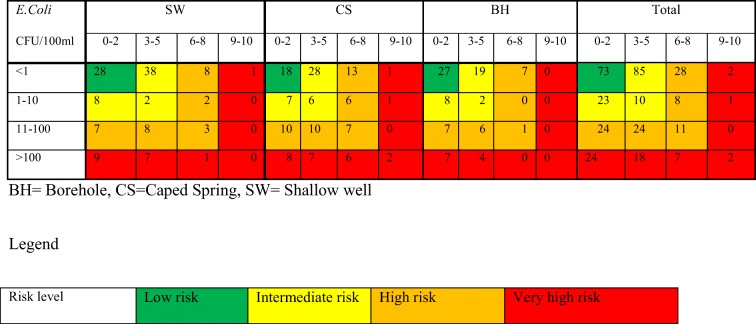
Risk priority matrix adapted from (WHO, 2012)

### Microbial water quality

All 340 water samples collected were analyzed for the indicator organisms *E. coli* and enterococci. The water quality analysis result for improved water source (boreholes, protected shallow wells, and caped springs) is summarized according to percentage of water samples and their microbial load in Table 1. The microbial load was characterized into different categories based on the risk levels. Samples with *E. coli* and/or enterococci < 1 CFU/100 ml are in conformity with WHO guideline, 1–10 CFU/100 ml with low risk, 10–100 CFU/100 ml with intermediate risk, 100–1000 with high risk, and > 1000 CFU/100 ml with very high risk (WHO, 2012). For the simplicity of interpretation of results, the risk level of enterococci was also categorized as *E. coli*.

The density of *E. coli* and enterococci differs greatly among sources from a minimum of 0 to a maximum of 7500 and 9876 CFU/100 mL, respectively. Of the 340 water samples for all combined water sources, 188 (55.3%) samples for *E. coli* and 167 (49.1%) for enterococci complied with the WHO guideline (0 CFU/100 ml). However, 152 (44.7%) and 173 (50.9%) of the samples were contaminated with *E. coli* and/or enterococci respectively, at different levels of contamination. Of the contaminated samples, 152 (44.7%) were positive for both *E. coli* and enterococci while 21 (6.2%) of the sources contained enterococci only.

The water quality differences by water source types according to the microbial risk level of *E. coli* and enterococci for boreholes, caped springs, and protected shallow wells are shown also in Table 1. More than half, i.e., 53 (60.2%), of the samples taken from boreholes were at the level with no risk of contamination *E. coli* < 1 CFU/100 ml. The remaining 35 (39.8%) samples were at low- to intermediate-risk levels and 11 (12.5%) at high and very high risk of contamination. From caped spring sources, more than 50% of the samples did not comply with WHO guideline value. Most of the samples 27 (20.7%) were at intermediate risk of *E. coli* from 10 to 100 CFU/100 ml. Out of the shallow wells, 75 (61.5%) met the WHO guideline value with no risk of contamination. The remaining 38.5% of the samples verified from low to very high risk. The more frequent value was 18 (14.7%) at intermediate level of contamination.

Of 88 boreholes, in 51 (58%) of the samples, enterococci were not detected in 100 ml. The contamination level differed per borehole, 28 (31.8%) of the water samples were at low- and intermediate-risk level, whereas 9 (10.2%) of the samples were at high-risk level. Very high risk (> 1000 count/100 ml) of enterococci was not observed for borehole samples. From caped spring sources, only 46 (35.4%) of the samples were enterococci free. Higher incompliance was observed in this source; 84 (64.6%) were contaminated with enterococci at different microbial risk levels, 60 (46.2%) were at low- and intermediate-risk, and 24 (18.4%) of the samples were at high- and very high-risk levels. From 122 protected shallow well samples analyzed, 70 (57.4%) were negative for enterococci, 41 (33.6%) of the samples were at low and intermediate risk while 11 (9%) were at high and very high risk.

### Sanitary inspection

The results observed from sanitary inspection of 340 sites were evaluated in different risk levels summarized in Table 2. Based on WHO guideline, it is considered low if the risk score is between 0 and 2; intermediate risk, 3 and 5; high risk, 6 and 8; and very high risk, greater than 8. From all types of water sources, 144 (42.4%) samples scored a low-risk level, whereas 137 (40.3%) scored an intermediate-risk, 54 (15.9%) a high-risk, and 5 (1.5%) a very high-risk level. The main risks identified at those unsafe water sources were that:

- the drainage canals were blocked with mud, grass, leaves, and stones;
- animals drinking the overflow water and grazing in the proximity of water sources;
- inadequate protection of water sources such as absence of fences and diversion ditches;
- stagnant water near the source;
- construction of latrines close to water source at less than 30-m radius and located upstream;
- Animal feces such as cow dung were observed inside the fence. Mostly, residents bring their animals to the water sources for drinking, and donkeys and horses are also taken to transport water, while posing risks of contamination.

To verify any correlation between *E. coli* and enterococci contamination with the sanitary inspection score, Spearman’s rank correlations were calculated. The sanitary score for each water source type and also for all combined sources did not correlate with microbial quality of water.

### Combined sanitary inspection and water quality result

The combined analysis of water quality data of *E. coli* with a sanitary inspection risk score, the so-called risk priority matrix (WHO 2012), was calculated for different water source types and presented in Table 3. The risk priority matrix showed that only 73 (21.5%) of the water sources were at low risk. The remaining sources were at risks from intermediate- to very high-risk level. One hundred eighteen (34.7%) of the samples were at an intermediate risk, 95 (27.9%) at high risk, and 54 (15.9%) of the samples were at very high risk. From those different water source types categorized as high and very high risk, 32 (9.4%) were boreholes, 46 (13.5%) were protected shallow wells, and 71 (20.9%) were caped springs.

## Discussion

Improved water sources are drinking water sources that are protected from the outside environment by concrete covers (WHO/UNICEF 2017). Improved sources include piped water, boreholes or tube wells, protected dug wells, caped springs, and packaged or delivered water (WHO/UNICEF 2017). It is believed that these water sources are safer compared to unimproved sources such as open wells and surface water. However, it is untrue that all improved sources are safe for drinking and other household purposes. The microbial water quality analyses showed that 45% of water samples taken from improved water sources were contaminated with *E. coli* and 51% of water samples were contaminated enterococci. This result is in agreement with Tadesse et al. (2010) who performed a rapid water quality assessment in Ethiopia from 2004 to 2005 and indicated that only 58.6% improved water sources respect the WHO guideline value for total coliform bacteria. In Dire Dawa City Administrative Council, Ethiopia, a bacteriological water quality analysis was conducted by Amenu et al. (2012) from protected springs and wells, and the result demonstrates that 85% of water samples taken were positive for indicator bacteria. In the Dominican Republic, the assessment of microbial quality of improved drinking water showed that the quality of water was poor with more than 90% of the water samples contaminated with *E. coli* bacteria and 47% of samples were having high and very high sanitary risk to human health (Baum et al., 2014).

Different reasons can be presented to justify the reason for the contamination of improved water sources in this study. In the SNNPR, the water supply from shallow wells and boreholes was not continuous; water was only collected in the morning and evening for two reasons. The first reason was that in the arid and semi-arid areas of the country, the water yield is minimal. To avoid the shortage of water, residents abstract water at a fixed time of the day especially in the dry season. The second reason is that motorized water sources function by diesel and electric power for the pumping of water from the ground. When there is interruption of electricity, water supply will be disrupted for unlimited hours in a day. This intermittent supply of water may lead to the contamination of water due to re-growth and multiplication of microbes. Besides, when water stays at a static condition and under negative pressure, microorganisms may be drawn from the surrounding environment into the water source (Ayoub, 2006). Tokajian and Hashwa (2003) confirmed in their study that drinking water quality was compromised due to re-growth of microorganisms associated with intermittent supply of water in Lebanon and could occur in the SNNPR.

The sanitary inspection score for each water source type and also for all combined sources did not correlate with microbial quality of water. This study agrees with Luby et al. (2008) and indicates that the sanitary condition does not necessarily predict the microbial quality of water and it is an indication that other routes of contamination of water sources are more important. On the other hand, our study contradicts with the investigation performed by Howard et al. (2003b) who showed a strong correlation between sanitary score and the presence of thermotolerant coliforms in spring water samples; this contradictory result may be due to the study period which coincided with the rainy season, whereas in our survey, most of our studies were conducted in the dry season. Emphasis has been given to seasonal variations on microbial contamination of water sources by different researchers. Godfrey et al. (2005) confirmed that the microbial concentration of water increased in rainy season than in the dry season. In this season, water sources can be contaminated through poorly maintained infrastructure as rain water collects pollutants while it runs off farms, streets, and waste disposal areas. In dry season, in most areas of the southern region, more droughts are expected so that this method may be replaced by a better method which suites the dry season.

From the sanitary inspections, one of the risks identified in the area was the closeness of pit latrines to the water sources (less than 30 m) situated uphill from the water source. In this situation, contaminants can be transported to the groundwater through fractured aquifers in the subsurface due to openings and cracks in the soil and rock (Graham & Polizzotto 2013).

From the three water source types, protected springs were more highly contaminated than boreholes and shallow wells. This result is in agreement with Tadesse et al. (2010); the water quality survey performed on improved water sources in different regions of Ethiopia indicated that spring water sources were more contaminated by total coliform bacteria in the SNNPR. This may be due to less effectiveness of the water treatment method. Because all spring water sources have no tap, the water flows continuously and very quickly. The added chlorine will be immediately washed away; it has no enough contact time to kill microorganisms in the water. On the contrary, chlorine introduction to boreholes and shallow wells has enough contact time for disinfection and therefore, the water will be well-treated before being pumped out and distributed to consumers.

## Conclusion

The study conducted in the Southern Nations, Nationalities, and Peoples’ Region has clearly indicated that people may be at risk of being exposed to pathogens in half of the improved water sources when used for drinking based on the microbial indicator data or the sanitary inspection risk score.

Though no correlation resulted from the water quality and sanitary condition of sources, risk priority matrix did enable prioritization of 54 very high-risk-level water sources for targeted interventions from a total of 340 improved drinking water sources. However, the survey was performed and sources only sampled once and such a study could be repeated to inform decision makers of progress. Moreover, most of the samples were collected in the dry season explaining the absence of the sought correlation. It is very apparent that the rainy season has more impact on the quality of water due to water movement with a possibility of ingression of contaminants into the water sources. To ensure comprehensive results, it is very appropriate to extend the study to surveying the water sources in the rainy season and more frequent analysis of the microbial water quality. Moreover, strengthening of water supply management could be further improved by so-called climate resilient water safety planning.

From this, targeted interventions could be recommended, such as improving the construction of water sources, safeguarding the water sources by preventing animals, flood, and any contaminants entering in to the facilities, identifying and implementing effective water treatment options, providing sustainable energy sources for the supply of continuous water, and establishing national water quality surveillance and monitoring systems to verify that the water being supplied to the consumer remains to be safe and adequate all the time. Those interventions can help to prevent diarrheal diseases such as cholera from relapsing in the country every year.

## Abbreviations

BH: Borehole
CS: Caped spring
CFU: Colony-forming units
*E. coli*: *Escherichia coli*
ISO: International Standard Organization
SDG: Sustainable development goal
SNNPR: Southern Nations, Nationalities, and Peoples’ Region
SW: Shallow well
UNICEF: United Nations Children’s Fund
WHO: World Health Organization
